# Relationship between income and concerns about physical changes and help-seeking by older adult cancer survivors: a secondary analysis

**DOI:** 10.1186/s12877-023-03887-1

**Published:** 2023-03-29

**Authors:** Irene Nicoll, Gina Lockwood, Fay J. Strohschein, Lauren Fitch, Christopher J. Longo, Lorelei Newton, Margaret I. Fitch

**Affiliations:** 1 Healthcare Consultant, Toronto, Canada; 2 Biostatistician Consultant, Toronto, Canada; 3 Faculty of Nursing, University of Calgary, Calgary, AB Canada; 4 Physiotherapist, Riverview, New Brunswick, Canada; 5 Health Policy and Management, DeGroote School of Business, McMaster University, Hamilton, ON Canada; 6 School of Nursing, University of Victoria, Victoria, BC Canada; 7 Bloomberg Faculty of Nursing, University of Toronto, 207 Chisholm Ave, M4C 4V9 Toronto, ON Canada

**Keywords:** Cancer survivors, Finances/income, Survivorship care, Aged, Help-seeking, Rehabilitation

## Abstract

**Objective:**

Globally, the number of older adults surviving cancer is anticipated to grow rapidly over the next decades. Cancer and its treatment can leave survivors with a myriad of challenges including physical changes which impact independence and quality of life. This project explored the relationship of income level with concerns and help-seeking for physical changes following treatment in older Canadian survivors of cancer.

**Methods:**

A Canada-wide survey of community-dwelling survivors of cancer explored their experiences with survivorship care one to three years following completion of treatment. A secondary trend analysis examined the relationship of income with older adults’ level of concern and help-seeking experiences regarding physical consequences they attributed to their cancer treatment.

**Results:**

In total, 7,975 people aged 65 years and older who survived cancer responded to the survey, of whom 5,891 (73.9%) indicated annual household income. Prostate (31.3%), colorectal (22.7%) and breast (21.8%) cancer accounted for the majority of respondents. Of those who reported household income data, over 90% wrote about the impact of physical changes following treatment, their concerns about the changes, and whether they sought help for their concerns. The most frequently identified physical challenge was fatigue (63.7%). Older survivors with low annual household incomes of less than $CA25,000 reported the highest levels of concern about multiple physical symptoms. 25% or more of the survey respondents across all income levels reported difficulty finding assistance for their concerns about the physical challenges, especially in their local communities.

**Conclusion:**

Older survivors of cancer can experience a range of physical changes, amenable to intervention by physical therapy, yet experience challenges obtaining relevant help. Those with low income are more severely affected, even within a universal healthcare system. Financial assessment and tailored follow-up are recommended.

## Introduction

The number of survivors of cancer is expected to exceed 20 million worldwide by 2025, [1, 2] with older adults being one of the fastest growing cohorts within this population [1]. Over the next decade, the number of people diagnosed with cancer who are 65 and older will double, reaching levels of 14 million new cancer diagnoses worldwide and accounting for over half (60%) of all new cancer cases [3].

A diagnosis of cancer and its treatment can leave survivors with physical (e.g., fatigue, pain), emotional (e.g., depression, fear of recurrence), and practical (e.g., transportation, financial) consequences which influence their quality of life and capacity [4]. Over 50% of Canadian adults aged 61 or older have two or more high impact, high prevalence chronic conditions [5], consistent with observations in other countries [6, 7]. Some have adaptive capacity to withstand stressors of a cancer experience, whereas others have decreased resilience and are less able to return to baseline after treatment [8, 9]. In most cancer care settings, survivorship care is not considered part of cancer treatment services. Post-treatment clinical appointments with cancer specialists are often brief for survivors leaving little time for discussion and assessment of a full range of concerns. These factors add to the challenge of obtaining assistance for concerns that survivors may be experiencing.

In general, cancer survivors report a myriad of unmet physical, psychosocial, and practical needs following the completion of their cancer treatment [4]. Physical changes following treatment have been described as frequent and major challenges by older survivors of cancer [10]. Fatigue, pain, weakness, joint stiffness, and balancing and walking difficulties can impose physical limitations on older survivors, restricting their daily living and return to pre-treatment activities. Access to physical therapy, occupational therapy, pre-habilitation, rehabilitation, and exercise programs can assist in recovery of physical changes; [11, 12] however, referrals to these programs are not standardized for survivors of cancer and barriers to accessing the services are reported [2]. One barrier may be financial.

Although Canada has one of the lowest poverty rates for seniors in the world, 12.5% of older Canadians are living in poverty [5]. Furthermore, employment income is the main source of income for over 40% of Canadian seniors 65 years of age or older, suggesting employment may be a necessity, particularly for those without private retirement income [13]. Older adults are frequently on fixed incomes and may not have access to financial resources such as savings and investment income [14]. Those no longer working are not likely participating in employee health benefit plans or may not have personal health insurance coverage [15]. In addition, older adults can experience a decreased social support network (e.g., loss of friends/family members, children living at a distance) and need to pay for services such as transportation, homecare, and assistance with activities of daily living.

Since the early 2000s, dealing with financial challenge has been reported as an unmet need for people with cancer in high income countries, even with universal healthcare systems [16], and unmet financial needs have been shown to increase with age [17]. For survivors, between 28% and 48% report experiencing financial burden due to direct (e.g., out-of-pocket expenses) and indirect (e.g., lost income) costs [16]. Older adults are frequently on fixed incomes and may not have access to financial resources such as savings and investment income [27]. Not having finances to manage cancer treatment symptoms and side effects during or after treatment, may impact long-term effects in survivorship and quality of life [18].

Therefore, the aim of this paper is to explore the relationship between income and concerns regarding physical changes and help-seeking among people aged 65 years and older after completion of cancer treatment, by conducting secondary analysis of a publicly available dataset from a national survey. Gathering insight into the concerns older survivors have about these concerns has not previously been reported for such a large Canadian sample nor linked to an income perspective.

## Methods

The full description and rationale for the Transitions Survey Study is reported in a previous publication [19]. In brief, this survey, *Experience of Cancer Patients in Transition*, was designed by oncology experts based on a conceptual framework regarding needs of survivors of cancer and subjected to face and content validity testing by healthcare providers and survivors. It was mailed in 2016 to a randomly selected sample of 40,790 community-dwelling survivors of cancer from across all ten Canadian provinces. The survey targeted survivors between one- and three-years following completion of primary cancer treatment. The eligibility criteria included adults (age 30 + years) treated for breast, prostate, colorectal, and melanoma cancers with no metastatic spread, and selected hematological (e.g., Hodgkin’s lymphoma, diffuse B cell lymphoma, acute myelogenous leukemia, acute lymphocytic leukemia) cancers; as well as adolescents and young adults (AYA, 18 to 29 years) with all non-metastatic cancer types except testes, where metastatic disease was included (see respondent profile in Table 1).

**Table 1:** Respondent Profile (N = 5,891)

Variable	Number	Percentage
Income †		
- < 25,000 - 25,000 to < 50,000 - 50,000 to < 75,000 - > 75,000	1,198 2,214 1,237 1,242	20.3 37.6 21.0 21.1
Sex		
- Male - Female - No answer	3,473 2,400 18	59.0 40.7 0.3
Age		
- 65–74 - 75–84 - 85 and older	3,577 1,914 400	60.7 32.5 6.8
Marital Status		
- Single - Married/partnered - Separated/divorced/widowed - Prefer not to answer	266 4,156 1,423 46	4.5 70.5 24.2 0.8
Education		
- High School or less - Post-secondary degree (college/university) - University graduate degree - Missing	4,360 854 558 119	74.0 14.5 9.5 2.0
Disease site*		
- Prostate - CRC - Breast - Melanoma - Hematological - Other - Missing	1,844 1,335 1,286 618 425 189 385	31.3 22.7 21.8 10.5 7.2 3.2 6.5
Metastases		
- No metastases - Living with metastases - Unsure - Missing	4,494 527 516 354	76.3 8.9 8.8 6.0
Time since treatment		
- < 1 year - 1 year to < 3 years - 3 years or more - Did not receive treatment - Missing	751 2,525 1,453 941 221	12.7 42.9 24.7 16.0 3.7
General physical health		
- Very poor/poor - Fair - Good/very good - Missing	223 1,326 4,310 32	3.8 22.5 73.2 0.5
General emotional health		
- Very poor/poor - Fair - Good/ very good - Missing	166 917 4,481 327	2.8 15.6 76.1 5.6
Overall quality of life		
- Very poor/poor - Fair - Good/ very good - Missing	122 950 4,806 13	2.1 16.1 81.6 0.2
Comorbidities		
- Yes - No - Missing	4,123 1,563 206	70.0 26.5 3.5
Comorbidities (4 most common)		
- Cardiovascular or heart condition; hypertension or high blood pressure - Arthritis, osteoarthritis, or other rheumatic disease - Diabetes - Mental health issues	2,228 1,978 859 474	54.0 48.0 20.8 11.5

† 2,084 of 7,975 (26.1%) of adults aged 65 and older are excluded from this analysis because income data was missing

*Percentages add to greater than 100 because respondents could select multiple sites

The original survey was designed to assess experiences of survivors of cancer who were most apt to be followed in the community, identify their concerns following cancer treatment, and explore their experiences in transitioning to follow-up care [19]. The survey was implemented to understand experiences of community-dwelling survivors as they the transition from cancer treatment to survivorship care, defined as care given to patients after finishing primary cancer treatment and prior to identification of recurrent disease.

As part of the survey, respondents indicated their concerns about physical challenges by choosing from a list of nine potential changes, derived from relevant survivorship literature. The changes, reflecting those most likely to be reported by survivors, included fatigue, pain, peripheral neuropathy, sexual function, bladder/bowel problems, gastrointestinal problems, lymphedema, cognitive changes and hormonal changes [20]. For each physical change experienced, respondents rated their degree of concern *(‘big’, ‘moderate’, ‘small’, ‘not a concern’)*, whether they had sought help (*Yes/No*), and if they had sought help, how easily help was obtained (‘*very easy’, ‘easy’, ‘hard’, ‘very hard’ and ‘didn’t get any help’*). Examples for a range of potential types of help were included as illustration. If they did not seek help for a concern, they were asked to indicate why by choosing from a list of pre-set reasons including an open-ended option. Respondents were also asked to indicate whether they received useful information about physical concerns and/or if they were required to find information on their own (using a scale of ‘*strongly agree’, ‘somewhat agree’, ‘agree’, ‘disagree’, ‘somewhat disagree’, strongly disagree’ and ‘not applicable’)*. As part of demographic information, respondents were asked to report their total annual household incomes before taxes. Annual household income was collected as a categorical variable with five levels (in Canadian dollars): < $25,000; $25,000 to $49,999; $50,000 to $74,999; $75,000 to $124,999; and > $125,000.

Ethics approval was granted by respective ethics boards in the ten provincial cancer agencies participating in survey distribution. Participants signed consents prior to completing the survey. The national survey data are housed in a publicly available platform.

### Analysis

For this secondary analysis, data were extracted from the publicly available national survey database [19] for all respondents 65 years and older. Analysis was performed for those older respondents who answered the income question and focused on their responses regarding the physical change questions.

The frequencies of individual concerns were calculated for the nine physical domain items and corresponding levels of concern. The income categories $75,000 to $124,999 and greater than $125,000 were collapsed to present a four-category variable, based on the notion that those in the upper income groups would have greater access to disposable income than those in the lower groups. For help-seeking, a category ‘*difficulty getting help’* was created including responses ‘*hard*’, ‘*very hard’*, and *‘did not get help’*.

For each of the physical change questions, prevalence of concerns, help-seeking, and difficulty getting help, as well as reasons for not seeking help and responses to information questions, were examined for trends across income groups. Crosstabulations of each variable by income groups were presented and proportions were assessed for patterns across the ordered income groups. Specifically, the proportions were tested using Cochran-Armitage tests to determine if they increased or decreased as the income level increased (referred to as an increasing or decreasing trend over income). The Cochran-Armitage test was used as this method assesses a stronger question as to whether the differences are increasing or decreasing over the ordered categorical variable. Data were analyzed using SAS v.9.4. P-values < 0.05 were considered significant.

## Results

### Demographic characteristics

The national survey [19] had a 33% response rate (n = 12,929) including 7,975 respondents 65 years or older who are the focus of this secondary analysis. Of these older respondents, 5,891 (73.9%) answered the demographic question about annual household income. Just over 20% (n = 1,198, 20.3%) reported income less than $25,000; 37.6% (n = 2,214) $25,000 to under $50,000; 21.0% (n = 1,237) $50,000 to under $75,000; and 21.1% (n = 1,242) over $75,000. 59% of these respondents were male and over 50% were married or had partners. Those between the ages of 65 and 74 represented 60.7% of respondents, those 75 to 84 32.5%, and those 85 and older 6.8%. 74% indicated they had achieved high school education or less. Overall demographic characteristics are presented in Table 1.

### Concerns about physical changes following treatment

The majority of older respondents (between 88.7% and 92.8%) answered the questions about concerns regarding the nine physical changes listed. Over 63% of those who responded indicated concerns with fatigue/tiredness. Over 40% reported concerns with changes in sexual activities/functions and bladder or urinary problems, and over 30% reported concerns with gastrointestinal problems, changes in concentration/memory and nerve problems (see Table 2). Significant trends over income groups were evident for all the concerns except bladder or urinary problems (see Table 2). In general, the trends were decreasing, indicating that the lower income groups had the higher percentages of concerns. The single exception was for changes in sexual activities/function, where the percentage reporting a concern was lower for the under $25,000 income group.

**Table 2:** Prevalence of concerns about physical changes by size of concern

Physical Changes	Number (%) of respondents indicating a concern about the change (small, moderate or big)					Number of concerns reported that were rated small				Number of concerns reported that were rated moderate				Number of concerns reported that were rated big			
	*< 25 K*	*25-50 K*	*50-75 K*	*75 + K*	P *	*< 25 K*	*25-50 K*	*50-75 K*	*75 + K*	*< 25 K*	*25-50 K*	*50-75 K*	*75 + K*	*< 25 K*	*25-50 K*	*50-75 K*	*75 + K*
**Fatigue/** **tiredness**	759 (71%) N=1,069	1,350 (66%) N=2,044	715 (61%) N=1,171	660 (56%) N=1,184	< 0.001	163 (21%)	387 (29%)	227 (32%)	245 (37%)	305 (40%)	578 (43%)	322 (45%)	271 (41%)	291 (38%)	385 (29%)	166 (23%)	144 (22%)
**Changes in sexual activities/** **function**	342 (33%) N=1,049	842 (41%) N=2,044	541 (46%) N=1,170	553 (47%) N=1,187	< 0.001	77 (23%)	180 (21%)	121 (22%)	133 (24%)	81 (24%)	256 (30%)	190 (35%)	172 (31%)	184 (54%)	406 (48%)	230 (43%)	248 (45%)
**Changes in concentration or memory**	418 (40%) N=1,052	666 (33%) N=2,013	319 (27%) N=1,163	302 (26%) N=1,170	< 0.001	181 (43%)	319 (48%)	164 (51%)	171 (57%)	157 (38%)	248 (37%)	115 (36%)	100 (33%)	80 (19%)	99 (15%)	40 (13%)	31 (10%)
**Nerve problems**	386 (37%) N=1,050	659 (33%) N=2,008	350 (30%) N=1,162	285 (24%) N=1,170	< 0.001	131 (34%)	267 (41%)	166 (47%)	119 (42%)	140 (36%)	238 (36%)	126 (36%)	114 (40%)	115 (30%)	154 (23%)	58 (17%)	52 (18%)
**Chronic pain or long-term pain**	419 (41%) N=1,027	607 (30%) N=2,006	277 (24%) N=1,155	237 (20%) N=1,169	< 0.001	132 (31%)	255 (42%)	118 (43%)	112 (47%)	172 (41%)	228 (38%)	99 (36%)	90 (38%)	115 (27%)	124 (20%)	60 (22%)	35 (15%)
**Hormonal, menopause or fertility**	180 (18%) N=986	375 (19%) N=1,947	214 (19%) N=1,135	178 (15%) N=1,155	0.05	49 (27%)	110 (29%)	74 (35%)	60 (34%)	61 (34%)	137 (37%)	77 (36%)	66 (37%)	70 (39%)	128 (34%)	63 (29%)	52 (29%)
**Gastrointestinal problems**	456 (44%) N=1,043	780 (38%) N=2,030	393 (34%) N=1,165	380 (33%) N=1,168	< 0.001	148 (32%)	272 (35%)	148 (38%)	151 (40%)	165 (36%)	293 (38%)	153 (39%)	135 (36%)	143 (31%)	215 (28%)	92 (23%)	94 (25%)
**Bladder or urinary problems**	423 (40%) N=1,053	847 (42%) N=2,023	464 (40%) N=1,169	485 (41%) N=1,184	0.91	132 (31%)	289 (34%)	177 (38%)	183 (38%)	145 (34%)	333 (39%)	173 (37%)	179 (37%)	146 (35%)	225 (27%)	114 (25%)	123 (25%)
**Lymphedema**	289 (28%) N=1,021	419 (21%) N=1,991	184 (16%) N=1,155	145 (13%) N=1,164	< 0.001	99 (34%)	168 (40%)	70 (38%)	66 (46%)	112 (39%)	165 (39%)	72 (39%)	42 (29%)	78 (27%)	86 (21%)	42 (23%)	37 (26%)

* P-values are from the Cochran-Armitage test for trend across income categories

Overall, the proportion of respondents reporting ‘*big*’ concerns decreased as income increased. The highest percentages of concerns were observed for those in the under $25,000 income group who reported ‘*big*’ concerns related to changes in sexual activities/function (53.8%), hormonal, menopause or fertility (38.9%), fatigue/tiredness (38.3%), bladder or urinary problems (34.5%) and gastrointestinal problems (31.3%). In contrast, those in the highest income group reported significantly lower percentages regarding ‘big’ concerns related to these issues; changes in sexual activities/function (44.8%), hormonal, menopause or fertility (29.2%), fatigue/tiredness (21.8%), bladder or urinary problems (25.3%) and gastrointestinal problems (24.7%).

### Help-seeking for concerns and difficulty experienced

There were significant trends across the income groups in the percentages of respondents with concerns who sought help for six of the nine physical changes. The percentage who sought help decreased as the income level increased. Percentages of those in the lowest income group who sought help were higher than those in the highest income group for fatigue/tiredness, changes in concentration or memory, nerve problems, gastrointestinal problems, and chronic and long-term pain (see Table 3). The exception was about changes in sexual activities/function where the percentages of those in the highest income group who reported a concern and sought help were higher.

**Table 3:** **Access to help for concerns regarding physical changes after cancer treatment** P-values are from the Cochran-Armitage test for trend across income categories

Physical changes	% of those with a concern about a physical change who sought help					% of those who sought help for their concern that experienced difficulty (hard or very hard to find help/no help obtained)				
	*< 25 K*	*25-50 K*	*50-75 K*	*75 + K*	*P*	*< 25 K*	*25-50 K*	*50-75 K*	*75 + K*	*P*
**Fatigue/** **tiredness**	297 (43%) N = 697	448 (36%) N = 1,258	198 (29%) N = 674	185 (29%) N = 631	< 0001	104 (36%) N = 291	159 (36%) N = 443	72 (36%) N = 198	61 (33%) N = 183	0.67
**Changes in sexual activities/** **function**	122 (39%) N = 316	302 (38%) N = 795	199 (38%) N = 521	239 (45%) N = 535	0.04	47 (39%) N = 120	120 (40%) N = 299	68 (34%) N = 198	85 (36%) N = 238	0.26
**Changes in concentration or memory**	115 (31%) N = 370	154 (25%) N = 615	62 (20%) N = 303	50 (18%) N = 282	< 0.001	63 (56%) N = 113	70 (47%) N = 149	29 (48%) N = 61	25 (51%) N = 49	0.46
**Nerve problems**	187 (53%) N = 351	329 (53%) N = 624	158 (47%) N = 334	131 (47%) N = 276	0.05	75 (41%) N = 184	128 (39%) N = 326	72 (46%) N = 156	53 (41%) N = 130	0.59
**Gastro-intestinal problems**	284 (66%) N = 429	471 (63%) N = 742	226 (60%) N = 377	216 (60%) N = 362	0.03	87 (31%) N = 280	113 (24%) N = 465	64 (28%) N = 225	67 (31%) N = 213	0.65
**Bladder or urinary problems**	249 (63%) N = 396	470 (58%) N = 817	267 (60%) N = 444	272 (58%) N = 473	0.28	65 (26%) N = 248	85 (18%) N = 463	61 (23%) N = 265	63 (23%) N = 272	0.94
**Chronic pain or long-term pain**	258 (64%) N = 401	326 (58%) N = 567	149 (56%) N = 268	112 (50%) N = 224	< 0.001	87 (34%) N = 254	108 (33%) N = 323	56 (38%) N = 148	41 (37%) N = 112	0.47
**Hormonal, menopause or fertility**	81 (47%) N = 171	148 (42%) N = 351	86 (42%) N = 205	78 (46%) N = 171	0.81	37 (47%) N = 79	50 (34%) N = 145	29 (35%) N = 84	27 (35%) N = 77	0.18
**Lymphedema**	176 (64%) N = 277	229 (57%) N = 404	112 (62%) N = 180	87 (61%) N = 142	0.87	43 (25%) N = 174	56 (25%) N = 228	30 (27%) N = 112	23 (26%) N = 87	0.66

Trends across the income groups in the percentages of those who sought help for their concern and expressed difficulty (i.e., ‘*hard’ or ‘very hard’ to ‘find help/no help obtained’*) were not significant (see Table 3). Between 18% and 56% of respondents across all income groups who sought help reported having difficulty finding assistance. 30% or more of the respondents across income groups reported difficulty finding help with six changes: concentration or memory (50.8%), nerve problems (41.2%), sexual function (37.4%), hormonal/fertility issues (37.1%), fatigue (35.5%) and chronic or long-term pain (34.9%).

Reasons for not seeking help were explored in each of the four income category groups (see Table 4). There was a significant increasing trend over income groups in the percentage choosing the reason, ‘*Told it was normal/thought nothing could be done’* where 37.0% of the highest income group (more than $75,000) reported this reason compared to 26.5% of the lowest income group (less than $25,000). Although selected less frequently, there were significant decreasing trends in percentages endorsing the following options: ‘*Did not think services were available’, ‘Did not know where to go’*, and ‘*I was embarrassed’* and ‘*Did not know I could ask’.* Lower income groups had higher percentages.

**Table 4:** Reasons for NOT seeking help regarding concerns about physical changes following cancer treatment by cancer survivors 65 + years

Reason	< 25 K (n = 642) +	25-50 K (n = 1,244)	50-75 K (n = 752)	75 + K (n = 714)	P
Told normal/thought nothing could be done	170 (26.5%)	386 (31.0%)	234 (31.1%)	264 (37.0%)	< 0.001
Did not want to ask	54 (8.4%)	118 (9.5%)	46 (6.1%)	58 (8.1%)	0.25
Did not think services were available	61 (9.5%)	82 (6.6%)	38 (5.1%)	44 (6.2%)	0.01
Did not know where to go	61 (9.5%)	61 (4.9%)	36 (4.87%)	26 (3.6%)	< 0.001
I was embarrassed	53 (8.3%)	73 (5.9%)	31 (4.1%)	29 (4.1%)	< 0.001
Did not know I could ask	23 (3.6%)	35 (2.8%)	14 (1.9%)	8 (1.1%)	0.001
Other*	316 (49.2%)	609 (49.0%)	413 (55.0%)	349 (48.9%)	0.48

+ Number of respondents reporting not seeking help for at least one concern who answered question about reasons

* Other category lumps together all the other responses that were not part of the six pre-set responses which offered a pre-set reason. Written responses were often unique to individual’s circumstances/perspectives

P-values are from the Cochran-Armitage test for trend across income categories

### Information about physical concerns

The percentage of respondents who somewhat or strongly disagreed with the statement, *‘I received useful information about my physical concerns’*, did not differ significantly over income groups and varied between 5.6% and 7.4% (see Table 5). The percentage of respondents who somewhat or strongly agreed with the statement, *‘I had to search for information on my own about my physical concerns’*, increased as income level increased. The highest income group (more than $75,000) was higher (26.1%) than the other three income groups (20.5%, 20.0% and 20.4% respectively).

**Table 5:** Unmet information needs* experienced by cancer survivors 65 + years

Information statement	< 25 K (n = 968)**	25-50 K (n = 1,809)	50-75 K (n = 1,023)	75 + K (n = 999)	P
I received useful information about physical concerns (somewhat or strongly disagree)	71 (7.3%)	102 (5.6%)	70 (6.8%)	74 (7.4%)	0.52
I had to find information on my own about my physical concerns (somewhat or strongly agree)	198 (20.5%)	361 (20.0%)	209 (20.4%)	261 (26.1%)	0.002

*Analysis is restricted to respondents who had at least one concern regarding a physical change

**Note that the n for each income category does not correspond to number with at least one concern in Table 2 because of missing data in the information variables

P-values are from the Cochran-Armitage test for trend across income categories

## Discussion and conclusion

This exploratory secondary analysis offers insights into relationships between income and concerns and help-seeking regarding physical changes as experienced by older Canadian adult survivors of cancer. The sample includes a cross-section of individuals from across Canada as well as various cancer types and income levels. Significant trends were observed across income levels with survivors in the lowest income groups reporting the highest percentage of concerns about physical changes and help-seeking for those concerns. However, older survivors across all income levels reported difficulty obtaining relevant help for their concerns.

This is one of the first studies to specifically explore the influence of income for older adult survivors of cancer and their perspectives regarding physical concerns. The specific challenges regarding access to services which could assist with physical challenges following cancer treatment may be most applicable to the Canadian situation, given variations in geography and funding of the healthcare system [15]. However, the insights could be applicable for similar contexts and the types of challenges experienced by older Canadian survivors may inform investigations in other countries.

Strong trends were evident across income levels with survivors in the lowest income level reporting a significantly higher proportion of ‘*big*’ concerns regarding physical changes in contrast to those in the highest income level. Higher proportions of individuals in low-income groups having higher numbers of concerns has been reported by other investigators [21]. Concerns such as fatigue/tiredness, pain, and bladder and nerve problems, commonly reported by survivors of cancer [22], all have important impacts on physical mobility, functional status and a person’s ability to engage in basic and instrumental activities of daily living, particularly among older adults [23]. Availability of help for these key physical limitations could have a critical impact on the ability of older survivors to maintain a satisfactory quality of life and level of independence during and after cancer treatment, outcomes of great significance to them [24]. The implications of having physical changes may be greater for older survivors who continue to work, either by choice or necessity, and for those in lower income levels who do not have the financial means to hire help at home or pay for services to assist them. These physical concerns are amenable to intervention and access to relevant assistance could be beneficial for recovery of older survivors.

The percentage of respondents who sought help decreased as income levels increased. This is not entirely surprising given that respondents in the higher income levels may be more apt to have access to assistance without requiring resources outside the home. However, the concerning observation is the sizeable proportion of respondents who sought help, regardless of income group, and experienced difficulty obtaining it. This raises questions about accessibility and availability of interventions, services, and programs for older survivors of cancer overall.

While some physical changes were not adequately addressed according to some survey respondents in this sample, the reasons may be specific to perspectives about the change itself. Healthcare providers and patients may discount the changes as being associated with aging [25], and cancer care providers may be unprepared to assess these issues [26]. For example, concentration/memory issues may be considered as expected for seniors and cancer care providers may be unprepared to manage cognitive changes. Issues with sexuality can be underestimated for this population by care providers, resulting from expectations and attitudes related to ageism [27]. The changes that were not adequately addressed could also imply there are system barriers such as the lack of availability and/or access support programs for these issues and/or seniors’ knowledge of or capacity to participate in them. Cancer care providers may view these issues as outside the realm of cancer care and may lack of knowledge about community-based rehabilitation services which can provide needed assistance with physical changes. Finally, poor coordination and communication with primary care providers may delay referral and service provision [28].

More than a quarter of respondents across income levels who did not seek help for their concerns selected the reason, ‘*Told it was normal/thought nothing could be done’*. It is not clear who informed individuals about this perspective or why they held this viewpoint. This may be associated with physical changes being associated with aging, by healthcare providers and by patients themselves [25]. However, this reason for not seeking help should be of concern to healthcare professionals as physical changes can be identified and interventions exist that could alleviate discomfort and promote independence [11, 12]. Individuals do not need to struggle on their own to manage these issues; however, they do need to be aware of the services which are available in the community for them and how to self-refer as needed.

Use of currently available screening tools (e.g., Edmonton Symptom Assessment Scale [29, 30], Canadian Problem Checklist [31]) and integration of geriatric assessment tools into survivorship care planning would advance the capacity of healthcare providers, whether in cancer centres or in primary care environments, to assess the risk and potential impact of physical changes following cancer treatment on age-related areas of concern [32]. Such tools would also facilitate the identification of risk for financial burden. Healthcare providers need to be familiar with the physical changes following cancer treatment experienced by older survivors, assessment or management of these changes and help-seeking patterns of older adults. They also need to be knowledgeable about relevant resources available in the community setting, the expertise of professional allied health groups such as physiotherapy, occupational therapy. and kinesiology, referral pathways for appropriate rehabilitation services, how best to inform survivors about the benefits of intervention, and available financial supports. Gaps have been identified regarding communication, referral and coordination of survivorship care between cancer specialist centres and primary care practitioners in the community setting [28].

Finally, it is important to understand more about how financial status, and especially what constitutes financial distress for an individual, may shape the experience of help-seeking by older survivors of cancer. This type of information could assist healthcare professionals from cancer centres and primary care settings to assess needs of older survivors and craft survivorship care plans tailored across the income spectrum for this population. It also highlights the need to include social workers as part of the cancer care team.

### Implications

The study found significant trends across income groups for ‘*big*’ concerns, and help-seeking, suggesting low-income groups are at higher risk. While the topic of finances can be uncomfortable for both survivors and healthcare professionals to discuss, it is important to recognize the importance of addressing financial concerns and their relationship to accessing necessary assistance for physical changes following treatment. Screening and decision-making tools to engage older patients around issues of cost as well as symptom management, and structures and processes around assessment and management of financial distress at the institutional level, are needed [33]. Communication and conversations about financial burdens are important, including follow-up with relevant interventions as needed (e.g., referral to financial support resources, referral to social work, financial counsellors, support groups for free or inexpensive transportation to appointments) [2, 15]. In addition, healthcare systems should ensure survivors across all income levels know about, and have access to, existing programs, information, and resources about physical changes they could experience and what can be done to reduce the challenges.

### Limitations

Several limitations exist with this analysis. Confidentiality issues limited information about survivors that could be shared from the registry, leaving insufficient detail to allow weighting of survey results to have them representative of all Canadian survivors of cancer. Further, although the intention of sampling was to target five disease sites and survivors one to three years post-treatment, self-reported survey data revealed that just under 10% of survivors indicated they had a cancer type outside the five targeted originally, only 55.6% of older adults reported being between one to three years post-treatment, and 76.3% indicated not having metastases. As noted, 23.4% of older adults did not disclose their household income and there was no way to assess whether the missing data are random over the income groups.

Additionally, the sample does not reflect the income distribution across Canada of those aged 65 years and older [34]. Low-income populations and non-English/French speakers are underrepresented. Hence, results cannot be generalized to the Canadian population of those aged 65 years and older at large.

This secondary analysis was an exploration using a publicly available data set, thus imposing limitations in the variables available for incorporation into this work. Analysis of income alone is not sufficient to explain the variations in levels of concern and help-seeking. Future analysis would benefit from including educational and occupational data and incorporating other social determinants of health.

Finally, the measure of income was objective, asking about annual household income, and may not directly correspondent or reflect perceived financial difficulty. Older individuals could have considerable savings or investments yet low household income. It also does not account for the number of people in the household or whether the amount identified in the survey was a decrease from before the cancer diagnosis. Perceived financial difficulty may also be associated with the region in which one lives, as cost of living may vary by geographic region as does availability and costs of specific healthcare services.

## Conclusion

Healthcare professionals need to recognize the impact of physical changes following cancer treatment for older individuals as they transition to survivorship and the impact of income level on their concerns about physical changes, help-seeking, and the likelihood of receiving assistance regarding their concerns. In general, there are higher percentages of concerns regarding physical changes and help-seeking among those with lower levels of income. These exploratory findings emphasize that further research is needed regarding associations between income and access to various support services for older adult survivors of cancer. Importantly, challenges in receiving help and information to support management of physical concerns were seen across all income levels which suggests systemic processes require review and improvement.

## Data Availability

Canadian Partnership Against Cancer has full control of primary unidentifiable record level data. The dataset is publicly available (https://www.systemperformance.ca/transition-study/).
